# Evidence for Concurrent Effects of Exposure to Environmental Cadmium and Lead on Hepatic CYP2A6 Phenotype and Renal Function Biomarkers in Nonsmokers

**DOI:** 10.1289/ehp.7192

**Published:** 2004-07-28

**Authors:** Soisungwan Satarug, Muneko Nishijo, Pailin Ujjin, Yuvaree Vanavanitkun, Jason R. Baker, Michael R. Moore

**Affiliations:** ^1^National Research Centre for Environmental Toxicology, University of Queensland, Brisbane, Australia; ^2^Department of Public Health, Kanazawa Medical University, Uchinada, Ishikawa, Japan; ^3^Department of Laboratory Medicine, Chulalongkorn University, Bangkok, Thailand; ^4^Department of Clinical Pharmacology, Flinders University, Adelaide, Australia; ^5^Queensland Health Scientific Services, Brisbane, Australia

**Keywords:** β-2-microglobulin, cadmium, coumarin, cytochrome P450 2A6, drug-metabolizing enzyme, environmental exposure, lead, liver drug metabolism, *N*-acetyl-β-d-glucosaminidase, nicotine, nicotine C-oxidase, proteinuria, zinc

## Abstract

We examined the interrelationships between phenotype of hepatic cytochrome P450 2A6 (CYP2A6), nephropathy, and exposure to cadmium and lead in a group of 118 healthy Thai men and women who had never smoked. Their urinary Cd excretion ranged from 0.05 to 2.36 μg/g creatinine, whereas their urinary Pb excretion ranged from 0.1 to 12 μg/g creatinine. Average age and Cd burden of women and men did not differ. Women, however, on average showed a 46% higher urinary Pb excretion (*p* < 0.001) and lower zinc status, suggested by lower average serum Zn and urinary Zn excretion compared with those in men. Cd-linked nephropathy was detected in both men and women. However, Pb-linked nephropathy was seen only in women, possibly because of higher Pb burden coupled with lower protective factors, notably of Zn (*p* < 0.001), in women compared with men. In men, Pb burden showed a negative association with CYP2A6 activity (adjusted β= −0.29, *p* = 0.003), whereas Cd burden showed a positive association with CYP2A6 activity (adjusted β= 0.38, *p* = 0.001), suggesting opposing effects of Cd and Pb on hepatic CYP2A6 phenotype. The weaker correlation between Cd burden CYP2A6 activity in women despite similarity in Cd burden between men and women is consistent with opposing effects of Pb and Cd on hepatic CYP2A6 phenotypic expression. A positive correlation between Cd-linked nephropathy (urinary *N*-acetyl-β-d-glucosaminidase excretion) and CYP2A6 activity in men (*r* = 0.39, *p* = 0.002) and women (*r* = 0.37, *p* = 0.001) suggests that Cd induction of hepatic CYP2A6 expression and Cd-linked nephropathy occurred simultaneously.

The metals cadmium and lead are ubiquitous environmental pollutants of increasing worldwide concern because of their renal toxicity and long residence time in the kidney [International Programme on Chemical Safety (IPCS) 1992; Jarup et al. 1998; Madden and Fowler 2000; Satarug et al. 2000]. These metals are absorbed by the body via enteral and pulmonary routes from dietary sources, drinking water, and polluted air (Bhatnagar 2004; IPCS 1992; Galal-Gorchev 1993; Satarug et al. 2004b). Safe levels of dietary intake, known as the provisional tolerable weekly intake (PTWI), have been established for Cd and Pb (World Health Organization 1989). The PTWI for Pb is 25 μg/kg body weight/week, which is approximately three times more tolerated than Cd, with a PTWI of 7 μg/kg body weight/week. Cd accumulates in the liver and kidneys, whereas Pb preferentially accumulates in the bone (IPCS 1992; Satarug et al. 2002). Cd in liver and Pb in bone are mobilizable to the kidney, providing an opportunity for nephrotoxicity with no additional exposure. The manifestations of Cd nephrotoxicity include proteinuria, calciuria, aminoaciduria, glycosuria, and tubular necrosis (IPCS 1992; Jarup et al. 1998).

Chronic exposure to low-level Cd results in 20- to 40-fold higher Cd concentration in kidneys than in the liver. However, although liver Cd levels are much lower than kidney levels, suggestive evidence for liver effects of Cd comes from immunoblotting of postmortem liver samples with anti-peptide antibody preparations against various human drug-metabolizing enzymes of the cytochrome P450 (CYP) superfamily, which revealed correlation between tissue Cd contents and the abundance of certain CYPs (Baker et al. 2001, 2002, 2003). Pb effects on liver drug metabolism were reported in early studies, where diminished phenazone elimination rates in men who had been occupationally exposed to Pb and who had evidence of clinical Pb poisoning were reversed after the patients had been treated with EDTA chelation therapy (Fischbein et al. 1977; Meredith et al. 1977; Moore 2004). A primary effect of Pb that could cause depressed CYP-mediated phenazone metabolism was its reduction of heme bioavailability via Pb induction of hepatic expression of the enzyme heme oxygenase (Moore et al. 1987), which degrades heme (Tenhunen et al. 1968) and Pb inhibition of the enzyme δ-aminolevulinate (ALA) synthase of heme synthesis pathway (Moore et al. 1987). However, although Cd is a potent inducer of heme oxygenase (Alam et al. 1989, 2000; Rosenberg and Kappas 1991) and an inhibitor of ALA synthase (Fujita 1997), as is Pb, Cd also has the ability to induce hepatic and renal cytochrome P450 2A5 (CYP2A5) in mice (Abu-Bakar et al. 2004; Bartosiewicz et al. 2001; Urbenjapol et al. 2001). The ability of Cd to induce the murine CYP2A5, which is an ortholog of human cytochrome P450 2A6 (CYP2A6), is consistent with results obtained from a study in healthy human subjects, which found a positive correlation between Cd burden and accelerated rate of hepatic coumarin metabolism known to be mediated exclusively by CYP2A6 (Satarug et al. 2004a).

CYP2A6 is one of the genetically polymorphic enzymes, known to be expressed predominantly in the liver and to be an exclusive catalyst of a conversion of coumarin to 7-hydroxycoumarin (7-OHC; Oscarson 2001; Pelkonen et al. 2000; Raunio et al. 2001). CYP2A6 metabolizes also the tobacco alkaloid nicotine to cotinine, which is metabolized further by CYP2A6 to 3′-hydroxycotinine (Benowitz et al. 2003; Messina et al. 1997; Nakajima et al. 1996). CYP2A6 is therefore known also as nicotine C-oxidase, and its phenotype, reflected by urinary 3′-hydroxy-cotinine:cotinine ratio, showed a positive correlation with daily cigarette consumption (Benowitz et al. 2003). Other substrates of CYP2A6 include a number of pharmaceuticals (methoxyflurane, halothane, losigamone, valproic acid, letrozole, fadrozole, disulfiram, and tegafur), the caffeine metabolite 17-dimethyl-xanthine, the tobacco-specific nitrosamines, and the tobacco alkaloid nicotine. Individuals with the *CYP2A6* gene deficiency were found to be protected from smoking dependence. This has led to the hypothesis that the *CYP2A6* genetic polymorphism is a sole determinant of individuals’ smoking dependence. Although conflicting results have been reported, casting doubt on such role of the *CYP2A6* genetic polymorphism (Tricker 2003). We hypothesize that CYP2A6 phenotypic variability seen among most healthy individuals is caused by their exposure to environmental substances, which have the ability to induce or suppress the hepatic *CYP2A6* gene expression.

Evidence for inducibility of hepatic CYP2A6 comes from studies showing that the metals cobalt and tin, and drugs and compounds of diverse structures such as phenobarbital, rifampicin, clofibrate, pyrazole, thioacetamide, and griseofulvin increase expression of murine CYP2A5, which is an ortholog of human CYP2A6 (Donato et al. 2000; Oscarson 2001; Pelkonen et al. 2000; Raunio et al. 2001). Some of these chemicals were indeed found to induce also CYP2A6 expression in primary culture human hepatocytes (Donato et al. 2000). Previously, we found that hepatic CYP2A6 activity was elevated in individuals who had high Cd burden (Satarug et al. 2004a), assessed by urinary Cd excretion, which is a widely used indicator of long-term Cd exposure (Lauwerys et al. 1994; Mueller et al. 1998). However, in the previous study both smokers and nonsmokers were included, which reflected mixed sources of exposure and heterogeneity in exposure pattern that may also have altered CYP2A6 phenotypic expression. Further, neither Pb exposure, zinc status, nor markers of renal tubular toxicity were determined for the previous subjects. The present study therefore was conducted on another group of subjects who had never smoked, to examine the association between increased hepatic CYP2A6 activity and dietary exposure to Cd. Another purpose was to explore the potential effects of Pb exposure, sex, age, and Zn and iron status, assessed by serum Zn and ferritin levels, on liver CYP2A6 phenotypic variability and renal toxicity.

## Materials and Methods

### Studied subjects.

This study was approved by the Institutional Ethical Committee on Human Experimentation, Chulalongkorn University Hospital, Faculty of Medicine, Bangkok, Thailand. The studies sample group consisted of 118 individuals (65 women, 53 men), 21–57 years of age. All subjects were non-smokers, and all were Thai nationals who took part in the study after giving informed consent. All subjects lived in residential areas surrounding Bangkok when the study was undertaken. Subjects were students, factory workers, teachers, and laborers. Frequency distribution of various occupations in men and women was similar. None of them had been exposed to Cd in the workplace, and the group was considered to represent the middle-class Thai population in socioeconomic status.

### Biologic sample collection.

One 15-mL blood sample was collected by venipuncture from each subject 1 hr after swallowing of one 15-mg coumarin tablet (Venalot, Schaper and Brummer GmbH and Co. KG, Salgitter, Germany) with 300 mL drinking water after overnight fasting. Aliquots of the blood samples collected were subject to routine hematology and clinical biochemistry analysis using the blood analysis automated system of the Chulalongkorn University Hospital. Serum ferritin levels were assayed by an immunoelectrochemiluminescence method (Boehringer Mannheim Elecsys 1010; Boehringer Mannheim and Roche Diagnostics, Bangkok, Thailand). Urine samples were also collected for 3 hr after Venalot administration. The urine samples from each subject were pooled and total urinary volume was recorded. One 5-mL aliquot of urine from each subject was analyzed for creatinine and routine urinalysis (pH, specific gravity, protein, glucose) within the day of collection. The remainder of the urine samples, together with aliquots of whole blood and serum samples, were stored at −80°C for later analysis.

### Analysis of biomarkers and metals.

Urine samples were analyzed for 7-OHC, Cd, total protein, β2-microglobulin (β2-MG), and *N*-acetyl-β-d-glucosaminidase (NAG). For 7-OHC analysis, urine samples were treated with β-glucuronidase (250 U/mL; Sigma-Aldrich, Sydney, Australia) in 1.0 M acetate buffer (pH 5.0) at 37°C for 30 min. Concentrations of 7-OHC in the β-glucuronidase–treated samples were quantified by the high-performance liquid chromatography system with C_18_-reverse phase column and a fluorescence detector (Walters et al. 1980). Equivalent procedures were conducted for determination of 7-OHC concentration in serum samples.

Cd levels in urine samples were determined by inductively coupled plasma mass spectrometry. The accuracy and precision of our urine metal analysis were assessed by a simultaneous analysis of samples of the urine metal control Lyphochek (Bio-Rad, Sydney, Australia). The level 1 and level 2 urine metal controls refer to the controls with different concentrations of metals. The mean values for Cd and Zn in the level 1 control urine were 6.2 and 713 μg/L, respectively, and the mean values for the same metals in the level 2 control urine were 11.2 and 1,176 μg/L. Our analysis of the level 1 control urine samples (*n* = 18) gave mean ± SD values of 6.1 ± 0.2 and 662 ± 38 μg/L for Cd and Zn, respectively. The coefficient of variation for the corresponding metal was 2.5 and 5.6%. Our analysis of the level 2 control urine samples (*n* = 18) gave mean ± SD values of 11.4 ± 0.3 and 1,077 ± 65 μg/L for Cd and Zn. The coefficient of variation for the corresponding metal was 2.5 and 6.0%. A urinary Cd concentration of 0.05 μg/L was assigned to the urine sample found to contain Cd below the detection limit of the method.

Protein in urine samples was determined by turbidimetry after protein precipitation with EDTA and benzethonium chloride using the automated system (Roche/Hitachi 717; Boehringer Mannheim and Roche Diagnostics). Urinary β2-MG was determined by latex immunoagglutination method (LX test, Eiken β2MG-II; Eiken and Shionogi Co., Tokyo, Japan), and urinary NAG was assessed with an enzymatic rate colorimetric assay. Urine β2-MG and NAG assays were conducted with automated systems. Urinary Cd, protein, β2-MG, and NAG were adjusted for creatinine excretion, and both creatinine-adjusted values and total amount of each analyte excreted in 3 hr were used. Similar conclusions were reached with both values. However, in evaluations for effects of Cd exposure by correlation and regression analyses, total amounts of Cd and renal function biomarkers excreted in urine in 3 hr were used because they related more significantly did than creatinine-adjusted figures.

### Statistical analysis.

The Kolmogorov-Smirnov goodness of fit test was used to test for conformity to normal distribution of measured and base-10 logarithmically transformed data (see details in “Results”). To identify relationships between variables, the statistical tests used were chi-square testing of cross-tabulated data, Pearson’s correlation test or Spearman’s rank correlation, and multiple regression. We used the Student *t*-test or Mann-Whitney rank-sum test to determine statistical significance levels for male–female differences in mean values of tested variables. In multiple regression analyses, an indicator of liver effect (CYP2A6 catalytic activity) was entered as a criterion variable, whereas age and urinary Cd, Pb, and Zn excretion were entered as explanatory variables.

## Results

Table 1 shows age and blood and urine chemistry profiles of men and women separately. Average age was similar for men (37 years) and women (38 years). Most measured parameters were higher in men than in women: hemoglobin, hematocrit, red blood cell counts, blood urea nitrogen, serum ferritin, serum Zn, and plasma creatinine concentrations (*p*-values, < 0.001–0.005). Urinary Zn excretion in men was 27% higher than in women (*p* = 0.001), but men’s and women’s urinary Cd excretion rates were similar. This produces a greater mean urinary Zn:Cd ratio in men than in women (*p* = 0.001). Average values for serum Cd and Pb were similar in men and women. However, average urinary Pb and urinary total protein excretion rates were higher in women than in men (*p* < 0.001 for urinary Pb and *p* = 0.001–0.005 for urinary protein excretion). On average, urinary β2-MG, NAG excretion, and urinary 7-OHC excretion rates were similar in men and women.

**Figure 1:**
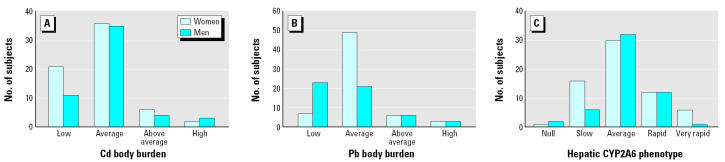
Frequency distribution of men and women across (*A*) Cd-burden groups, (*B*) Pb-burden groups, and (*C*) CYP2A6 phenotype groups, classed by percentile ranking. (*A*) Frequency distribution of men and women across Cd-burden group did not show statistically significant differences (likelihood ratio chi-squared = 2.6, *p* = 0.46). Cd-burden group was based on percentile ranking of rate of urinary Cd excretion: low, 5–30 ng/3 hr; average, 31–138 ng/3 hr; above average, 144–225 ng/3 hr; high, 235–360 ng/3 hr. (*B*) Difference in frequency distribution of men and women across Pb-burden group was statistically significant (chi-squared = 18, *p* < 0.001). Pb-burden group was based on percentile ranking of urinary Pb excretion: low, 0.01–0.14 μg/3 hr; average, 0.15–0.42 μg/3 hr; above average, 0.43–0.53 μg/3 hr; high, 0.54–1.01 μg/3 hr. (*C*) Null phenotype included three subjects (two men and one woman) who did not excrete any 7-OHC in the urine collected for 3 hr after dosing with 15 mg of coumarin. Frequency distributions of men and women across CYP2A6 phenotype groups tended to be different (likelihood ratio chi-squared = 7.3, *p* = 0.06). CYP2A6 phenotype group was based on percentile ranking of urinary excretion of 7-OHC in 3 hr after dosing with 15 mg of coumarin: null, 0 mg/3 hr; slow, 0.7–2.2 mg/3 hr; average, 2.5–6.8 mg/3 hr; rapid, 7.0–9.3 mg/3 hr; very rapid, 10.4–15.2 mg/3 hr.

The distribution of the logarithmically transformed Cd excretion rates conforms to normal (Kolmogorov-Smirnov goodness of fit test, *p* = 0.99). The geometric mean, median, and SD values were 54, 55, and 2.3 ng/3 hr, respectively. The lowest and highest urinary Cd excretion rates were 5 and 360 ng/3 hr, respectively. The overall frequency distribution of men and women in Cd-burden groups did not differ (likelihood ratio chi-squared = 2.6, *p* = 0.46; Figure 1A). The distribution of urinary Pb excretion rates conformed to normal, with mean, median, and SD values of 0.25, 0.23, and 0.18 μg/3 hr, respectively (Kolmogorov-Smirnov goodness of fit test, *p* = 0.17). The lowest and highest urinary Pb excretion rates were 0.01 and 1.06 μg/3 hr, respectively. The frequency distribution of men and women in Pb-burden groups differed significantly (likelihood ratio chi-squared = 18, *p* < 0.001; Figure 1B).

The distribution of urinary 7-OHC excretion rates was slightly skewed to the left of normal, with mean, median, and SD values of 5.06, 4.73, and 3.15 mg/3 hr, respectively (Kolmogorov-Smirnov goodness of fit test, *p* = 0.57). The highest urinary 7-OHC excretion rate was 15 mg/3 hr. Two men and one woman did not excrete 7-OHC, most likely because they lacked the *CYP2A6* gene or have a defect in the *CYP2A6* gene expression. The overall frequency distribution of men and women in CYP2A6 phenotype groups showed a tendency to be different (likelihood ratio chi-squared = 7.3, *p* = 0.06; Figure 1C). Of the seven subjects with a very rapid metabolizer phenotype of CYP2A6, six were women.

Table 2 summarizes the relationship between urinary Cd excretion, age, iron store status, and biomarkers of kidney function. We found no correlation between iron store status and Cd burden in men or women, but a strong positive correlation between urinary Cd excretion and age was seen in men (*r* = 0.47, *p* < 0.001). The correlation between urinary Cd and age was weaker in women, and it was not statistically significant (*r* = 0.20, *p* = 0.06). Both men’s and women’s urinary Cd excretion correlated positively with urinary protein excretion (overall *r* = 0.25, *p* = 0.003), but no correlation was seen between urinary Cd and β2-MG excretion in men or women. The correlation between urinary protein and Cd excretion in men and women remained statistically significant after controlling for age (partial *r* = 0.20, *p* = 0.02). Urinary NAG excretion rates showed a strong positive correlation with urinary Cd in both men and women (overall *r* = 0.49, *p* < 0.001). Controlling for age did not affect the strength of the NAG versus Cd correlation (partial *r* = 0.48, *p* < 0.001).

Table 3 shows results of equivalent correlation analysis with Pb exposure, age, and bio-markers of kidney function. Men’s urinary Pb excretion did not correlate with age or with any of the three renal biomarkers tested. Women’s urinary Pb excretion did not correlate with age, but it correlated with all three kidney toxicity markers, with the greatest correlation strength being between urinary Pb and NAG excretion (*r* = 0.50, *p* < 0.001), followed by urinary Pb and β2-MG excretion (*r* = 0.36, *p* = 0.002) and urinary Pb and protein excretion (*r* = 0.31, *p* = 0.01). We undertook partial correlation with controlling for Cd excretion because urinary excretion of the renal function biomarkers protein and NAG showed also significant correlations with urinary Cd (Table 2). The correlation between NAG and urinary Pb excretion was maintained (partial *r* = 0.39, *p* = 0.001) after controlling for Cd. The correlations between Pb and urinary protein (partial *r* = 0.09, *p* = 0.47) and between Pb and β2-MG excretion rates (partial *r* = 0.16, *p* = 0.19) both were lost after controlling for Cd.

Table 4 shows the dose–response relationship between Cd body burden and nephropathy as measured by increases in urinary excretion of NAG. The prevalence of subjects with abnormal urinary excretion of NAG (NAG-nuria) in low, average, above average, and high Cd body burden group was 3, 8, and 23%, respectively. Prevalence of subjects with NAG-nuria differed significantly across the three Cd body burden groups (linear trend chi-squared = 4.4, *p* = 0.04).

Table 5 shows the correlation matrix for CYP2A6 activity, age, urinary Zn excretion together with Cd, and Pb exposure in men and women, separately. Two men and one woman were not included in this correlation matrix because they did not excrete 7-OHC. Urinary Cd, Zn, and Pb correlated strongly with each other, with *r*-values of 0.55 and 0.53 in men and of 0.34 and 0.43 in women (*p* ≤0.001). Thus, partial correlation analysis was undertaken to control for Zn versus urinary 7-OHC excretion. In men, urinary 7-OHC excretion rates showed a positive correlation with urinary Cd (*r* = 0.32, *p* = 0.01), whereas it did not show any correlation with age or urinary Zn excretion. In addition, there was a significant inverse correlation between urinary 7-OHC and Pb (*r* = −0.32, *p* = 0.001) in men after adjusting for urinary Zn excretion. Multiple regression controlling for age and urinary Zn excretion confirmed a positive association between urinary Cd and 7-OHC excretion (adjusted β= 0.38, *t* = 2.9, *p* = 0.006) together with a negative association between urinary Pb and 7-OHC excretion (adjusted β= −0.29, *t* = 2.2, *p* = 0.003). As in men, women’s urinary 7-OHC excretion rates showed a positive correlation with urinary Cd (*r* = 0.21, *p* = 0.01), whereas it did not show any correlation with age or urinary Zn excretion. However, no correlation between urinary 7-OHC and Pb (*r* = 0.18, *p* > 0.05) was seen in women. Of note, women’s urinary Zn excretion showed an inverse correlation with age (*r* = −0.24, *p* = 0.01–0.05), but no such correlation was seen in men.

**Figure 2:**
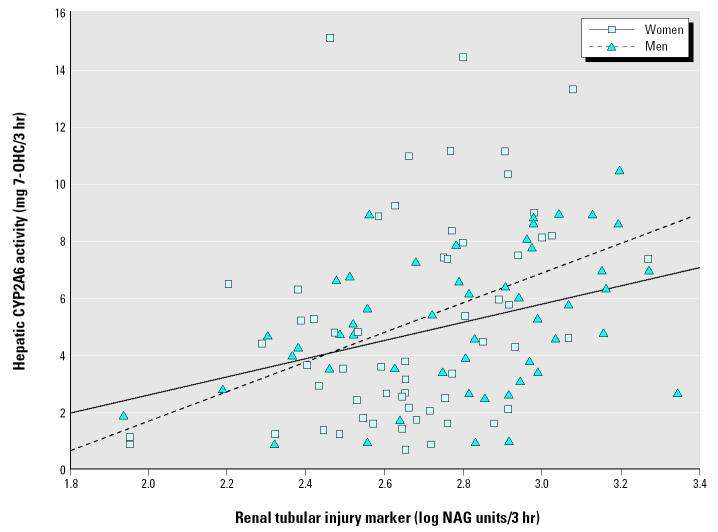
Pearson’s correlation analysis between rate of hepatic CYP2A6-mediated metabolism and the renal tubular toxicity marker NAG. The lines represent a positive correlation between the renal tubular toxicity bio-marker NAG excretion and hepatic CYP2A6 phenotype (urinary 7-OHC excretion after dosing with 15 mg of the probe xenochemical coumarin) in men and women, separately. The Pearson’s correlation coefficient *r*-values (*p*-value) for men and for women were 0.39 (*p* = 0.002) and 0.37 (*p* = 0.001), respectively.

Figure 2 shows a correlation between rates for hepatic CYP2A6-mediated coumarin metabolism and urinary excretion of the marker of renal tubular toxicity NAG in men and women, separately. The Pearson’s *r*-value for the correlation was 0.39 (*p* = 0.002) in men and 0.37 (*p* = 0.001) in women.

## Discussion

### Age- and Zn-related increment in Cd burden in men versus women.

A lack of an active biochemical process for Cd elimination coupled with renal reabsorption of Cd from the filtrate predicts age-related increment of Cd accumulation in various tissues and organs, notably liver and kidney (IPCS 1992; Jarup et al. 1998, Satarug et al. 2002). Thus, a positive correlation between subjects’ Cd burden and age is expected. Indeed, such expected correlation was observed with greater correlative strength and higher statistical significance in men (*r* = 0.47, *p* < 0.001) than in women (*r* = 0.20, *p* = 0.06). Body iron store status, however, did not show any correlation with women’s or men’s Cd burden, although a strong influence of body iron store status on Cd burden was shown previously in a group of women who were almost 10 years younger than the women in the present study (Satarug et al. 2004b). Thus, such discrepancy was most likely due to age-related differences in body iron status in women because it is known that women’s iron status is restored to normal at older ages (Custer et al. 1995). As with iron status, Zn is another important determinant of Cd burden and toxicity (Goyer 1997; Madden and Fowler 2000; Satarug et al. 2000). Thus, the correlation between age and Cd burden in women may be insignificant because the women’s Cd burden is more closely related to Zn status than iron status at older ages when their iron status becomes normal. Indeed, women’s urinary Zn excretion showed an inverse correlation with age (*r* = −0.24, *p* = 0.01–0.05; Table 5), but no such correlation was seen in men. We saw this as evidence for influence of Zn status on Cd burden. In addition, it provides an explanation for greater strength of the correlation between urinary Cd and Zn in men (*r* = 0.55, *p* < 0.001) than in women (*r* = 0.34, *p* = 0.001–0.005). A positive correlation between urinary Cd and Zn was expected in view of the fact that most of the Cd excreted in urine is bound to the low-molecular-weight metal-binding protein metallothionein together with Zn (Goyer 1997; Madden and Fowler 2000). Further research to show direct evidence for influence of Zn status on Cd burden and toxicity in women is required, and it should be focused on menopausal women because increased sensitivity to metal toxicity is noted in women (Nishijo et al. 2004; Vahter et al. 2002).

### Role of Zn in protection against Pb renal toxicity.

Women in the present study were more exposed to Pb than were men, as reflected by a 46% higher urinary Pb excretion rate in women than in men (*p* < 0.001; Table 1). Such Pb exposure levels experienced by these women appeared to have produced mild nephropathy, reflected by a significant correlation between urinary Pb and NAG, even after controlling for urinary Cd excretion (partial *r* = 0.39, *p* < 0.001). The Pb-linked renal effect seen here is consistent with literature reports (Madden and Fowler 2000) and a recent study where urinary NAG excretion was found to correlate positively with duration of Pb exposure, blood Pb, and nail Pb in policemen who were chronically exposed to the metal in polluted air (Mortada et al. 2001). In animal studies, Zn has consistently been shown to provide protection against Cd and Pb accumulation and toxicity (Furst 2002; Goyer 1997), but there is little research on such a role of Zn in humans. Our finding on the absence of evidence for Pb-linked renal toxicity in men was likely caused by lower Pb exposure levels coupled with higher protective factors in men than in women. Consistent with the protective role of Zn, men in the present study had 36% higher urinary Zn excretion together with higher Zn:Cd and Zn:Pb ratios than did women (*p* = 0.001–0.005).

### Concurrent renal and liver effects of Cd and Pb.

Cd exposure levels experienced by men and women in the present study were sufficient to cause a mild (subclinical) renal toxicity. This was suggested by a positive correlation between urinary Cd and NAG excretion rates in both men and women, after controlling for age (*r* ~ 0.5, *p* < 0.001). Renal tubular effects of exposure to low levels of environmental Cd detected in the present study confirmed data from population studies in Belgium (Buchet et al. 1990), China (Jin et al. 2002), Japan (Ikeda et al. 2000; Oo et al. 2000), the United States (Noonan et al. 2002), Sweden (Jarup 2002), and Thailand (Satarug et al. 2004a, 2004b). Further, a Cd-dose–dependent rise in the prevalence of abnormal urinary NAG excretion (NAG-uria) in subjects was shown (Table 4). The probability of having NAG-uria was increased by 20% as Cd burden increased from 0.05–0.26 μg/g creatinine to 1–2.36 μg/g creatinine.

We used rate of coumarin 7-hydroxylation as a marker of Cd and Pb liver effects because the reaction is known to be catalyzed exclusively by CYP2A6, which is expressed predominantly in the liver (Rautio et al. 1992; Satarug et al. 1996, 2004a; Ujjin et al. 2002). Evidence for liver Cd effect was suggested by a positive correlation between CYP2A6 activity and Cd burden seen in men and women, which remained statistically significant after controlling for age (Table 5). Such correlation agreed with the data in a previous study (Satarug et al. 2004a). However, despite overall similarity in Cd burden of subjects in the two sample groups, the strength of correlation between CYP2A6 activity and Cd burden was weaker in the present sample group (*r* = 0.21 in women and *r* = 0.32 in men) than in the previous group (*r* ~ 0.50 in men and women). This result was probably due to the smaller number of subjects combined with higher Pb exposure in the subjects in the present sample group than in the previous sample.

Evidence for Pb being a suppressor of liver CYP2A6 comes from a multiple regression analysis, controlling for Zn excretion and age, which revealed a negative association between urinary Pb and CYP2A6 activity (adjusted β= −0.29, *p* = 0.003) in men. The Pb suppressive effect on liver CYP2A6 seen here supports findings from previous reports on diminished phenazone metabolism in Pb-exposed workers (Fischbein et al. 1977; Meredith et al. 1977). In contrast with results in men, such inverse correlation between CYP2A6 activity and Pb exposure was not detected in women, although women showed higher urinary Pb excretion than did men. However, a weak correlation between CYP2A6 activity and Cd burden in women appeared consistent with Pb being a suppressor of liver CYP2A6 expression. The phenotype of hepatic CYP2A6 has been shown to be under the influence of liver pathophysiologic state, smoking, and exposure to a number of drugs and environmental factors (Kraul et al. 1991; Pasanen et al. 1997; Satarug et al. 1996, 2004c; Sotaniemi et al. 1995). Further, a recent study in 18 young male African green monkeys showed that exposure to nicotine at daily dose rates between 0.05 and 0.3 mg/kg for 18 days depressed catalytic activity of hepatic CYP2A6-like enzyme (Schoedel et al. 2003). It is therefore conceivable that a lack of an inverse correlation between Pb burden and CYP2A6 activity in women in the present study was more likely due to differences in dietary habits and exposure to the unidentified substances that can increase CYP2A6 expression, thereby offsetting the Pb effect.

Finally, evidence for concurrent effects of Cd in the liver and kidney comes from a positive correlation observed between the marker of kidney effect (urinary NAG excretion) and rate of hepatic CYP2A6-mediated metabolism (Figure 2). Highly statistical significance was observed for both men (*p* = 0.002) and women (*p* = 0.001), although the strength of correlation was modest in men (*r* = 0.39) and in women (*r* = 0.37). We further note that similar strength of correlation between urinary NAG excretion and CYP2A6 activity in men and women despite a weaker correlation between urinary Cd excretion and CYP2A6 activity in women than in men (Table 5). This may be caused by a very strong correlation between Cd concentrations in liver and kidney and between urinary NAG excretion and Cd concentration in the kidney, consistent with autopsy data, which showed that liver and urinary Cd concentration are closely related (Satarug et al. 2001).

## Conclusions

Dietary Cd and Pb exposure at the levels experienced by the subjects in the present study is associated with variation in hepatic CYP2A6 phenotypic expression and signs of mild nephropathy, detectable at the renal Cd concentrations of approximately 1–2 μg/g creatinine, corresponding to renal concentrations ≤50 μg/g kidney cortex. In women, Zn status may be an important determinant of Cd burden and Pb renal toxicity. In men, however, opposing effects of Pb (a suppressor) and Cd (an inducer) on phenotypic expression of hepatic CYP2A6 is observed. Thus, exposure to environmental Cd and possibly Pb may be implicated in the rate of nicotine metabolism because CYP2A6 is known to metabolize up to 90% of nicotine (Oscarson 2001; Pelkonen et al. 2000; Raunio et al. 2001). In addition, Cd and Pb exposure may explain differences among individuals in their reaction to therapy with certain drugs and their ability to handle a variety of environmental chemicals that are metabolized by CYP2A6. Associations between hepatic and renal effects of exposure to environmental Cd and possibly Pb observed in the present study imply that elevated CYP2A6 activity occurs in subjects who show signs of Cd-linked renal toxicity.

## Figures and Tables

**Table 1:** Age, blood, and urine chemistry profiles and assessment of Cd and Pb exposure and markers of liver and kidney effects.

Descriptor	Men	Women
No. of subjects	53	65
Age (years)	36.7 ± 9.4 (21–57)	38.1 ± 8.3 (23–55)
Body mass index (kg/m^2^)*^a^*	23.1 ± 3.0	22.4 ± 4.5
Hematological profiles		
Hemoglobin (g/dL)	14.5 ± 1.0	12.2 ± 1.1**
Hematocrit (%)	43.5 ± 2.7	37.1 ± 2.8**
Red blood cells (×10^6^/μL)	5.3 ± 0.5	4.4 ± 0.4**
White blood cells (×10^3^/μL)	7.4 ± 2.4	7.2 ± 1.9
Serum and urine chemistry profiles		
Ferritin (μg/L)	202 ± 161 (14–978)	60 ± 48 (3–197)**
Total protein (g/dL)	7.7 ± 0.5	7.7 ± 0.4
Plasma creatinine (mg/dL)	0.94 ± 0.12	0.66 ± 0.10**
Blood urea nitrogen (mg/dL)	12.6 ± 3.4	11.0 ± 2.5*
Serum Zn (mg/L)	1.38 ± 0.30	1.19 ± 0.16**
Urinary creatinine (mg/mL)	0.75 ± 0.71	0.61 ± 0.47
Urinary Zn excretion (μg/g creatinine)	371 ± 224	272 ± 164*
Cd and Pb exposure indicators		
Urinary Cd (μg/g creatinine)	0.48 ± 0.36 (0.05–1.6)	0.54 ± 0.39 (0.09–2.4)
Urinary Zn:Cd (mmol Zn/nmol Cd)	1.91 ± 1.82 (0.5 –13)	1.35 ± 1.14 (0.2–5)*
Urinary Pb (μg/g creatinine)	1.3 ± 1.8 (0.1–12)	2.4 ± 1.1 (0.6–6.8)**
Serum Cd (μg/L)	0.55 ± 0.48 (0.05 –2.5)	0.48 ± 0.44 (0.05–3.2)
Serum Pb (μg/L)	4.2 ± 5.4 (1–28)	3.0 ± 2.2 (1–12)
Kidney toxicity indicators*^b^*		
Protein (mg/g creatinine)	49 ± 47 (0.4 –121)	74 ± 67 (0.4–317)**
β2-MG (μg/g creatinine)	51 ± 121 (0.03–762)	29 ± 38 (0.03–218)
NAG (U/g creatinine)	4.4 ± 2.6 (0.6–15)	4.6 ± 2.2 (0.7–12)
Liver effect indicator		
Urinary 7-OHC (mg/3 hr)*^c^*	5.0 ± 2.6 (0–11)	5.1 ± 3.5 (0–15)

Values are mean ± SD; numbers in parentheses are ranges.

a Body mass index = body weight/square of height (kg/m^2^).

b Urine samples were collected for 3 hr after administration of 15 mg of coumarin.

c Two men and one woman did not excrete 7-OHC in urine, most likely due to their lack of the *CYP2A6* gene or having a defect in the *CYP2A6* gene expression.

* *p* = 0.001–0.005;

** *p* < 0.001.

**Table 2:** Correlation between urinary Cd excretion rates, age, iron store status, and markers of glomerular and tubular functions.

	Men		Women		All subjects	
Variables	*r*-Value	*p*-Value	*r*-Value	*p*-Value	*r*-Value	*p*-Value
Age	0.47*^a^*	< 0.001	0.20*^a^*	0.06	0.25*^a^*	0.003
Iron store status	−0.01*^a^*	0.46	0.17*^a^*	0.09	0.06*^a^*	0.26
Kidney function biomarkers						
Urine protein	0.23	0.05	0.30	0.01	0.27	0.003
Urine β2-MG	0.20	0.07	0.09	0.23	0.14	0.06
Urine NAG	0.51*^a^*	< 0.001	0.44*^a^*	< 0.001	0.49*^a^*	< 0.001

The *r*-values are the Spearman’s rank correlation coefficients unless otherwise specified. Significance of the correlation between each pair of variables in row and column is identified by the *p*-values ≤0.05.

a Pearson’s *r*-correlation coefficient value.

**Table 3:** Correlation between urinary Pb excretion rate, age, and markers of glomerular and tubular functions.

	Men		Women		All subjects	
Variables	*r*-Value	*p*-Value	*r*-Value	*p*-Value	*r*-Value	*p*-Value
Age	0.13*^a^*	0.17	−0.14*^a^*	0.13	0.02*^a^*	0.43
Kidney function biomarkers						
Urine protein	0.22	0.06	0.31	0.01	0.27	0.002
Urine β2-MG	0.12	0.19	0.36	0.002	0.21	0.01
Urine NAG	0.08*^a^*	0.27	0.50*^a^*	< 0.001	0.22*^a^*	0.007

The *r*-values are the Spearman’s rank correlation coefficients unless otherwise specified. Significance of the correlation between each pair of variables in row and column is identified by the *p*-values ≤0.05. The correlation between NAG and urinary Pb excretion was maintained (partial *r* = 0.39, *p* = 0.001) after controlling for Cd. The correlations between Pb and urinary protein (partial *r* = 0.09, *p* = 0.47) and between Pb and β2-MG excretion rates (partial *r* = 0.16, *p* = 0.19) were lost.

a Pearson’s *r*-correlation coefficient value.

**Table 4:** Dose–response relationship between Cd exposure and prevalence of NAG-nuria.

		Urine NAG (units/g creatinine)				
Percentile	Urinary Cd*^a^*	< 2.5	3–5	6–7	≥8	NAG-nuria (%)*^b^*
1st–25th	0.05–0.26	10	14	8	1	1/33 (3)
26th–90th	0.27–0.95	15	31	20	6	6/72 (8)
91st–100th	1.00–2.36	2	4	4	3	3/13 (23)

a μg/g creatinine.

b Urinary excretion of NAG ≥8 U/g creatinine, which indicates statistically significant differences in prevalence of subjects across urine–Cd and urine–NAG groups (linear trend chi-squared = 4.4, *p* = 0.04).

**Table 5:** Correlation matrix for rate of coumarin 7-hydroxylation, age, markers of renal tubular function, and Cd and Pb exposure.

Variables*^a^*	Urinary 7-OHC	Urinary Cd	Age	Urinary Pb	Urinary Zn
Male nonsmokers (*n* = 51)					
Urinary Cd	0.32*	1.00			
Age	0.09	0.47*^#^*	1.00		
Urinary Pb	−0.32*^b^*	0.19	0.13	1.0	
Urinary Zn	0.11	0.55*^#^*	0.21	0.53*^#^*	1.00
Female nonsmokers (*n* = 64)					
Urinary Cd	0.21*	1.0			
Age	−0.07	0.20	1.0		
Urinary Pb	0.18	0.39**	−0.14	1.0	
Urinary Zn	0.14*^c^*	0.34**	−0.24*	0.43*^#^*	1.0

Numbers are Pearson correlation coefficient (*r*) values unless otherwise specified.

a The correlation analysis did not include two males and one female who did not excrete 7-OHC in urine, most likely due to a lack of the *CYP2A6* gene or having a defect in the *CYP2A6* gene expression.

b Partial correlation between urinary 7-OHC and Pb after adjusting for urinary Zn.

c Partial correlation between urinary 7-OHC and Zn, after adjusting for urinary Pb.

* *p* = 0.01–0.05;

** *p* = 0.001–0.005;

# *p* < 0.001.
